# Time-variability of muscle oxygen saturation during graded maximal exercise

**DOI:** 10.1007/s00421-025-05871-6

**Published:** 2025-07-21

**Authors:** Lluc Montull, Natàlia Balagué, Monika Petelczyc, Karol Marszalek, Pablo Vázquez

**Affiliations:** 1 Complex Systems in Sport Research Group, National Institute of Physical Education of Catalonia (INEFC), University of Lleida, La Seu d’Urgell, Spain; 2 Complex Systems in Sport Research Group, National Institute of Physical Education of Catalonia (INEFC), University of Barcelona, Barcelona, Spain; 3 Cardiovascular Physics Group, Faculty of Physics, Warsaw University of Technology, Warsaw, Poland; 4 Faculty of Physics, Warsaw University of Technology, Warsaw, Poland; 5 Complex Systems in Sport Research Group, Autonomous University of Barcelona (UAB), Bellaterra (Cerdanyola del Vallès), Spain

**Keywords:** Time series analyses, Detrended fluctuation analysis, Sample entropy, Near infrared spectroscopy, Acute fatigue, Physiological networks

## Abstract

The time-variability of physiological and kinematic variables, extracted at mesoscopic and macroscopic levels, respectively, has shown potential in detecting changes in exercise workload and associated fatigue effects. However, the sensitivity of microscopic variables —such as muscle oxygen saturation, which reflect the dynamics of muscle metabolism—remains unexplored. This study aimed to compare the time-variability structure of the tissular saturation index (TSI) during a graded maximal exercise performed until exhaustion. Nineteen participants started running at 8 km/h with the speed increasing by 1 km/h every 100 s until they could not keep the prescribed velocity. The time-variability of TSI, recorded from the quadriceps, was analyzed using Detrended fluctuation analysis (DFA) and Sample entropy (SampEn) over the first and last 2048 recorded data points (corresponding to 204 s each). Wilcoxon test and Cohen’s d were used to compare the initial and final parts of the test. Results revealed a significant decrease in the Hurst (H) exponent (from H = 0.84 ± 0.21 to H = 0.49 ± 0.10; *p* < 0.01; *d* = -1.57) and a corresponding increase in SampEn (from 1.12 ± 0.20 to 1.40 ± 0.13; *p* < 0.01; *d* = 1.17). These findings indicate a shift towards uncorrelated white-noise as exhaustion approached, suggesting reduced efficacy of oxygen transportation with increasing workloads. The time-variability of muscle oxygen saturation appears to be a) a promising measure for assessing exercise intensity, and b) allow the study of physiological network interactions extracted from different levels (from microscopic to macroscopic).

## Introduction

The time-variability structure of certain physiological and kinematic variables, such as heart rate (HR), respiratory frequency, and accelerometry, has revealed its potential in detecting changes in exercise workload and associated fatigue effects (Billat et al. 2009; Gronwald et al. 2019; Van Hooren et al. 2023; Hunter et al. 2023; Montull et al. 2025; Rogers et al. 2025). Moreover, recent studies examining electromyography and electrocardiography time series have highlighted a loss of functional connectivity in intermuscular and cardiomuscular networks with accumulated effort (Garcia-Retortillo et al. 2023; Garcia-Retortillo and Ivanov 2024). This diminished network adaptivity is often associated with uncorrelated or rigid variability of the variables under study (Balagué et al. 2020; Pethick et al. 2021; Vázquez et al. 2016, 2021).

Muscle oxygen saturation is a key indicator of the balance between oxygen delivery and consumption within the muscles, providing valuable insights into metabolic dynamics during exercise (Barstow 2019). In recent years, near-infrared spectroscopy (NIRS) has emerged as a reliable, non-invasive tool for assessing muscle oxygen saturation, gaining significant attention in sports science and medicine (Oueslati et al. 2016; Jones et al. 2016; Lucero et al. 2018; Perrey 2022). NIRS has been proven effectiveness at different exercise intensities, showing higher values of muscle oxygenation in active muscles such as vastus lateralis (Kerhervé et al. 2017; Lucero et al. 2018; Manchado-Gobatto et al. 2020; Klusiewicz et al. 2021). The applications of this tool include, for example, estimating ventilatory and lactate thresholds (Sendra-Pérez et al. 2023; Batterson et al. 2023) or comparing vascular and metabolic profiles between highly active and sedentary populations (Tuesta et al. 2024).

Tissue haemoglobin saturation index (TSI), which reflects the percentage of oxygenated haemoglobin relative to total haemoglobin (oxygenated and deoxygenated), has been commonly recorded using NIRS. A decrease in TSI may indicate an imbalance between oxygen delivery and consumption during exercise (Mairbäurl 2013; Jones et al. 2015), as well as conditions such as exposure to altitude (Martin et al. 2009; Jeffries et al. 2019), anaemia (Crispin and Forwood 2021), or peripheral vascular diseases (Bauer et al. 2004; Mesquita et al. 2013). TSI and related NIRS-derived variables have shown particular promise for assessing task disengagement and cumulative effort, with responses differing across training levels in activities such as climbing (Feldmann et al. 2020), cycling (Zorgati et al. 2015; Yogev et al. 2023), or running (Oueslati et al. 2016). Although NIRS captures responses at the microvascular level, its variables are influenced by several integrated physiological mechanisms, including cardiac pump function, blood flow, respiratory rate, muscular metabolic capacity, and even muscle activation patterns (Daniel et al. 2023; Sendra-Pérez et al. 2023; Batterson et al. 2023; Tuesta et al. 2024). As such, muscle oxygen saturation can serve as a meaningful indicator of exercise workload.

Commonly applied measures or indices for studying muscle oxygen saturation typically focus on increasing–decreasing trends, peak values, and average distributions extracted from group-pooled data (Mairbäurl 2013; Perrey et al. 2024). This methodological approach is questioned by the Network Physiology of Exercise (Balagué et al. 2020, 2022), which emphasizes the need to detect individual patterns of response to exercise and generalize from individuals to the population, rather than the other way around. In the direction of personalized physiology, the proposed methodology suggests extracting individual time series of the variables under study (instead of their peak or threshold values), detect individual variability patterns in the time series, and analyzing the nonlinear dynamics to provide deeper insights into the response to exercise (Balagué et al. 2020).

In this regard, many studies have investigated the kinetics of muscle oxygenation using first-order dynamic models, typically based on mono-exponential functions that assume stationarity and linearity, to study for example how fast a muscle becomes deoxygenated during exercise or how quickly it reoxygenated (Ferreira et al. 2005; Boone et al. 2016; Perrey et al. 2024). However, while effective in quantifying response speed and amplitude, these approaches overlook the nonlinear and time-varying nature of physiological signals, limiting their sensitivity to detect changes of fluctuations. In contrast, nonlinear methods such as Detrended Fluctuation Analysis (DFA) and Sample Entropy (SampEn) can better capture the complexity and temporal variability of oxygenation signals—offering deeper insights into physiological adaptability—yet remain poorly explored to NIRS data during exercise.

DFA is a technique used to assess self-similarity of nonstationary time series. That is, whether time series exhibit positive autocorrelation (persistent dynamics), no correlation, or negative autocorrelation (anti-persistence) (Goldberger and West 1987; Peng et al. 1994, 1995; Ihlen 2012; Eke et al. 2012). The Hurst (H) exponent, derived from DFA, has proven to be a valuable outcome for assessing the response of physiological or kinematic variables in long-term scales (Eke and Hermán 1999; Galaska et al. 2008; Vázquez et al. 2016).

The H-exponent of 0.5 represents a signal characteristic of white noise or ordinary random walk, indicating no correlation or disorder in the studied variable (Hardstone et al. 2012). H values < 0.5 reflect negative correlations or anti-persistent time series, while values > 0.5 indicate positive correlations or persistent time series (Krstacic et al. 2002; Galaska et al. 2008). For physiological variables like Heart Rate Variability (HRV), increased exercise workload often reduces H values closer to 0.5, particularly in unhealthy populations, signifying a disordered and poor physiological response (Martinis et al. 2004; Aoyagi et al. 2005). Similar trends are observed in short-term correlation metrics such as DFA-α1 (Gronwald et al. 2020; Van Hooren et al. 2023; Rogers et al. 2025). Conversely, a persistent HRV profile, commonly observed during periods of rest or low-intensity exercise, reflects a more tightly regulated response, characterized by stable variability that indicates the capacity of the system to rapidly adjust to internal or external constraints (Gronwald and Hoos 2020; Van Hooren et al. 2023; van Rassel et al. 2025; Rogers et al. 2025).

Sample entropy (SampEn) is a widely used time-variability measure that quantifies the level of regularity or randomness in time series data (Richman and Moorman 2000; Delgado-Bonal and Marshak 2019). Similar to DFA, SampEn has primarily been applied to HRV and cardiovascular variables to detect physiological disorders and evaluate exercise responses (Lewis and Short 2007; Riganello et al. 2018). Jiang et al. (2021) extended its application to oxygen saturation, reporting higher SampEn values with increased exposure to hypoxia. Higher SampEn values indicate more unpredictable dynamics or greater randomness, which can be associated with a random walk or white noise behaviour in DFA (Hardstone et al. 2012). Greater entropy in oxyhemoglobin has also been found at cortical level when increasing the intensity of a cycling endurance exercise (Hong et al. 2024). Consequently, SampEn of oxygen saturation seems potential to carry information about the physiological response under increasing stress or workload. However, the time-variability of muscle oxygen saturation and the influence of exercise intensity—from baseline to voluntary exhaustion—remain underexplored, particularly in relation to the expected shift toward less predictable and more uncorrelated dynamics at higher intensities.

This study aimed to compare the time-variability structure of the TSI, particularly through the H-exponent and SampEn, between the beginning and the end of a graded maximal exercise. Our hypothesis was that due to increase of intensity and effort accumulation at the end of the exercise: i) the H-exponent of TSI will decrease towards an uncorrelated white noise (H ≈ 0.5), and ii) SampEn of TSI will increase towards less predictable dynamics.

## Materials and methods

### Participants

Nineteen university students of physical education (11 females, 8 males; 21.00 ± 2.29 yrs.; 1.71 ± 0.07 m; 64.57 ± 10.06 kg), who practiced sport regularly (7.78 ± 2.20 h/week), participated voluntarily in the study. They completed a questionnaire to confirm their health status. To estimate the sample size, a large effect size (ρ = 0.82), α = 0.05, and power (1-β) = 0.95 were determined. All experimental procedures were explained to participants before they gave their written consent to participate in the study. The experiment was approved by the Local Research Ethics Committee (004-CEICGC-2024) and carried out according to the Helsinki Declaration.

### Procedures

All participants, previously familiarized with the testing procedures, performed a graded maximal treadmill running test until voluntary exhaustion. The test started at 8 km/h and the velocity was increased by 1 km/h every 100 s until they could not keep the prescribed velocity. The rate of perceived exertion (RPE 6–20) scale (Borg 1998) and the HR via Polar H10 chest strap (Polar Electro Oy, Kempele, Finland) were recorded every 100 s, before changing the velocity. Consecutively prior to the running test, participants warmed up at 6 km/h for 5 min. After the test, participants walked at 6 km/h for 5 min to recover.

### Data acquisition

**Fig. 1:**
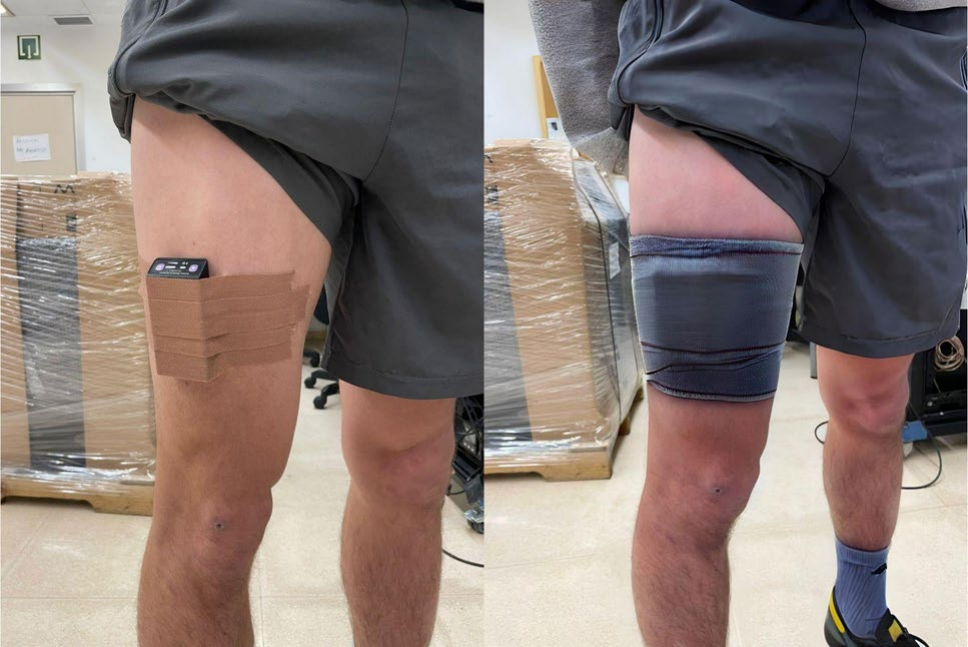
The PortaMon device was placed and secured on the vastus lateralis of the right leg

TSI was constantly recorded, from the warm-up to the end of the test, using the PortaMon device (Artinis Medical Systems®, Einsteinweg, Netherlands), which included three light source transmitters (each one with two wavelengths between 750 and 850 nm) placed at distances of 30, 35, and 40 mm from the receiver (Jones et al. 2014; Desanlis et al. 2024). As illustrated in Fig. 1, the device was placed on the belly of the vastus lateralis of the right quadriceps, midway between the greater trochanter of the femur and lateral femoral epicondyle (Jones et al. 2014). The sampling frequency was set at 10 Hz following the recommendations by Hesford et al. (2013) and Jones et al. (2014). To secure the probe and protect it from the environmental light, a dark band was tightly wrapped around the equipment (Manchado-Gobatto et al. 2020).

### Data analysis

We analyzed the temporal structure of the first and last 2048 raw data points of TSI time series, corresponding to 204 s each, through DFA and SampEn. Previously, the stationarity of each signal was formally assessed using the Augmented Dickey–Fuller (ADF) test. For signals where the null hypothesis of nonstationarity could not be rejected, an iterative procedure based on Empirical Mode Decomposition (EMD) was applied (Zhou et al. 2013). Specifically, the residual (global trend) was removed first, followed by successive removal of the lowest-frequency IMFs in ascending order of frequency, until the ADF test rejected the hypothesis about nonstationarity. Next the signal was normalized with mean and standard deviation of the stationary signal.

Finally, to support our findings, we performed the analysis of randomly shuffled original time series, in which correlations were destroyed but amplitude distribution preserved.

#### Detrended fluctuation analysis

DFA was performed as follows (according to Ihlen (2012) and Peng et al. (1995, 1994)): First, the total length of the TSI time series (N = 2048 data points) was integrated with the following equation:

$$Y(i)\equiv \sum_{k=1}^{i}\left[{x}_{k}- \langle x\rangle \right]$$

${x}_{k:}$ individual data point of the TSI time series.

$x:$ average TSI of the entire time series.

Then, the local trend was calculated to fit the TSI time series using a quadratic function (Ihlen 2012). Time series were divided into different windows (i.e., scales, with length *n* ranging from 20 to 200) (Hautala et al. 2003), in which the local trend was found. Obviously, the number of windows W depends on their length *n*: W_n_ = N/n. For successive *n* values, the magnitude of fluctuations was determined separately by substracting the local trend and constructing the final fluctuation function.

The fluctuation function was calculated in two steps. Using an integrated signal, the variance was determined in each window ν, ν = 1,…,W_n_ using the following equation (Kantelhardt et al. 2002):

$${F}^{2}\left(\nu ,n\right)=\frac{1}{n}{\sum }_{i=1}^{n}{\left\{Y\left[\left(\nu -1\right)\bullet n+i\right]-{y}_{\nu }(i)\right\}}^{2}$$

With.

*Y(i)*: integrated time series.

*y*_*ν*_*(i)*: local trend in each window.

Finally, fluctuation function F was defined by:

$$F\left(n\right)=\sqrt{\frac{1}{{W}_{n}}\sum_{\nu =1}^{{W}_{n}}{F}^{2}\left(\nu ,n\right)}={n}^{H}$$

The H-exponent, derived as the slope of the linear regression between the scale *n* and the local fluctuations in a log–log plot, was employed to characterize the nature of TSI fluctuations for each participant during the initial and final parts of the test. Matlab^©^ R2020 was used for this analysis.

#### Sample entropy

SampEn measures the likelihood that time series patterns (vectors) of the *m* length are close to each other and will remain close in the sequence of increased length *m* + 1 (Richman and Moorman 2000; Delgado-Bonal and Marshak 2019). Its calculation relies on the matching consecutive points within a tolerance *r* in the Chebyshev distance. To ensure reliable estimation of SampEn, a systematic optimization of the input parameters *m* (embedding) and *r* was performed. We explored a specified range for both input parameters to identify the configuration best suited to our dataset characteristics and signal dynamics. SampEn was calculated across *m* = 2,3,4 with *r* changes in increments of 0.05 starting from 0.1 up to 0.5. For each (*m, r*) pair, SampEn was computed separately for the initial and final parts of the test. To identify optimal parameters, we applied the following criteria that must be present in both phases: i) low inter-individual variability, defined as a coefficient of variation (CV = SD/mean) below 0.1; ii) robustness to changes in *r*, indicated by the a plateau-like SampEn values across increasing *r*, for a given *m*; iii) computational complexity – when multiple configurations satisfied the above conditions, a pair with the lowest *m* and *r* was selected. In result, *m* = 2 and *r* = 0.35 was found as optimal for TSI time series.

SampEn is defined by a negative logarithm, in which the average number of sequences under the tolerance is considered for both lengths *m* + *1* (in the numerator) and *m* (in the denominator). Low entropy values arise from regular physiological time series (like those with rhythmic fluctuations, Kosciessa et al. (2020)), and higher values reflect more random, irregular behaviour (Costa et al. 2005). Also, high values are typical for stochastic data sets (Lake and Moorman 2011; Weippert et al. 2014). Matlab^©^ R2020 was used for this analysis.

#### Statistics

Wilcoxon matched pairs test was used to compare average values, H-exponents and SampEn of TSI between the initial and final parts of the exercise. The significance level for the statistical tests was set at *p* = 0.05. Cohen’s d was estimated for the test statistics Z of the previous analysis to demonstrate the magnitude of standardized mean differences (Fritz et al. 2012). According to Cohen's (1988) guidelines, *d* ≥ 0.2, *d* ≥ 0.5, and *d* ≥ 0.8 represent small, intermediate, and large effect sizes, respectively. Statistical analyses were performed with SPSS v.15 (SPSS Inc., Chicago, USA).

## Results

The maximum velocity of the test was 15.74 ± 1.85 km/h, while the maximum HR was = 191.63 ± 6.55 bpm, and the maximum RPE was 19.01 ± 0.81. The mean values of HR and RPE from the initial and final 204 s, corresponding to the analyzed sections, was HR = 134.72 ± 9.83 bpm and RPE = 8.94 ± 1.17 at the beginning, and HR = 186.37 ± 6.28 bpm and RPE = 18.79 ± 0.73 at the end, respectively.

TSI average values largely decreased from the initial (68.24 ± 2.53%) to the final part (61.03 ± 2.50%) of the graded maximal running test (Z = -3.58, *p* < 0.01; *d* = -2.88).

**Fig. 2:**
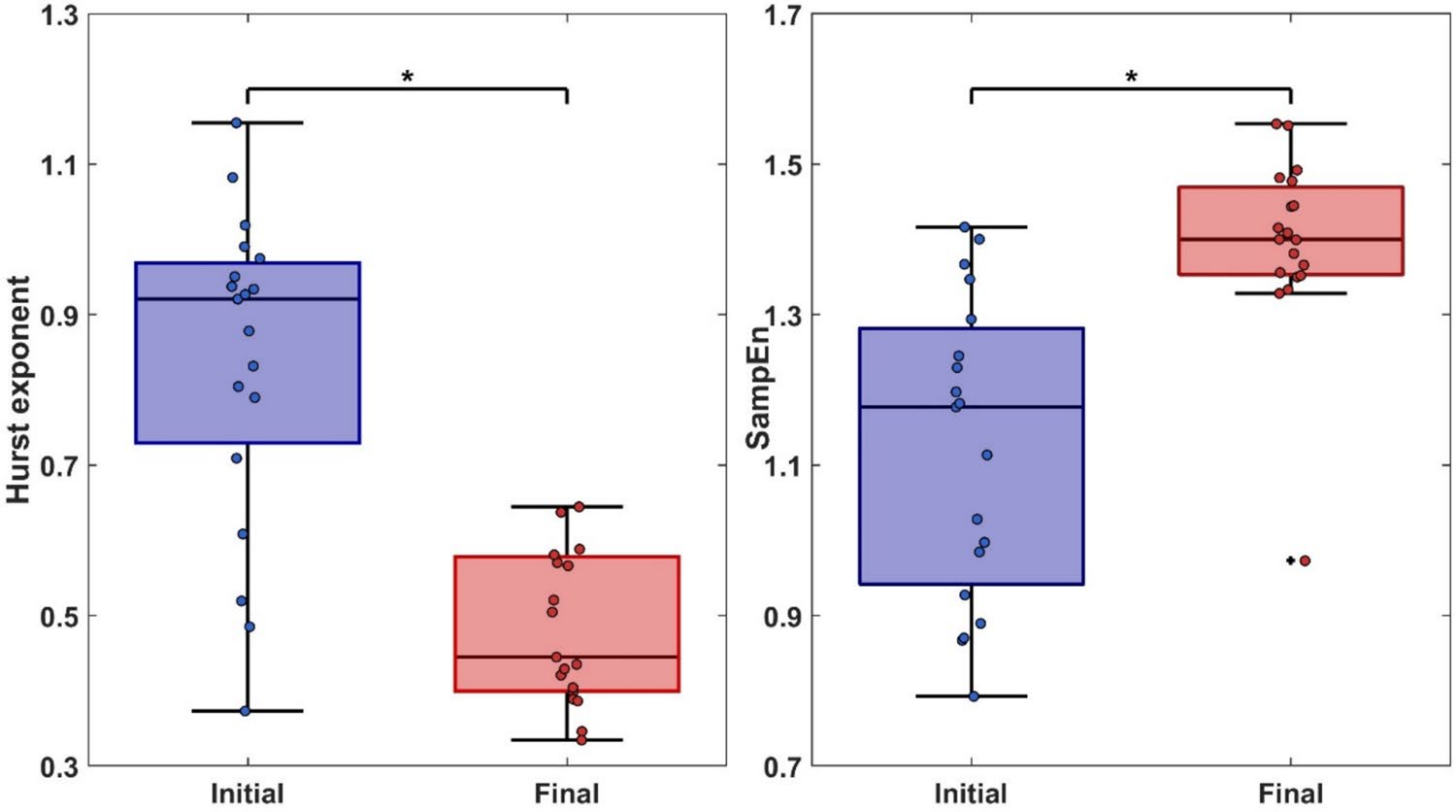
Box-plots illustrating the decrease of Hurst exponent (left) and increase of Sample entropy (SampEn) (right) of the Tissue saturation index (TSI) between the initial and final parts of the running test.

The comparison of the time-variability of TSI between the initial and the the final part was as follows (see Fig. 2):

- H-exponents largely decreased from H = 0.84 ± 0.21 at the beginning to H = 0.49 ± 0.10 at the end of the exercise (Z = -6.84, *p* < 0.01; *d* = -1.57).
- SampEn largely increased from 1.12 ± 0.20 at the beginning to 1.40 ± 0.13 at the end of the exercise (Z = -5.11, *p* < 0.01; *d* = 1.17).

**Fig. 3:**
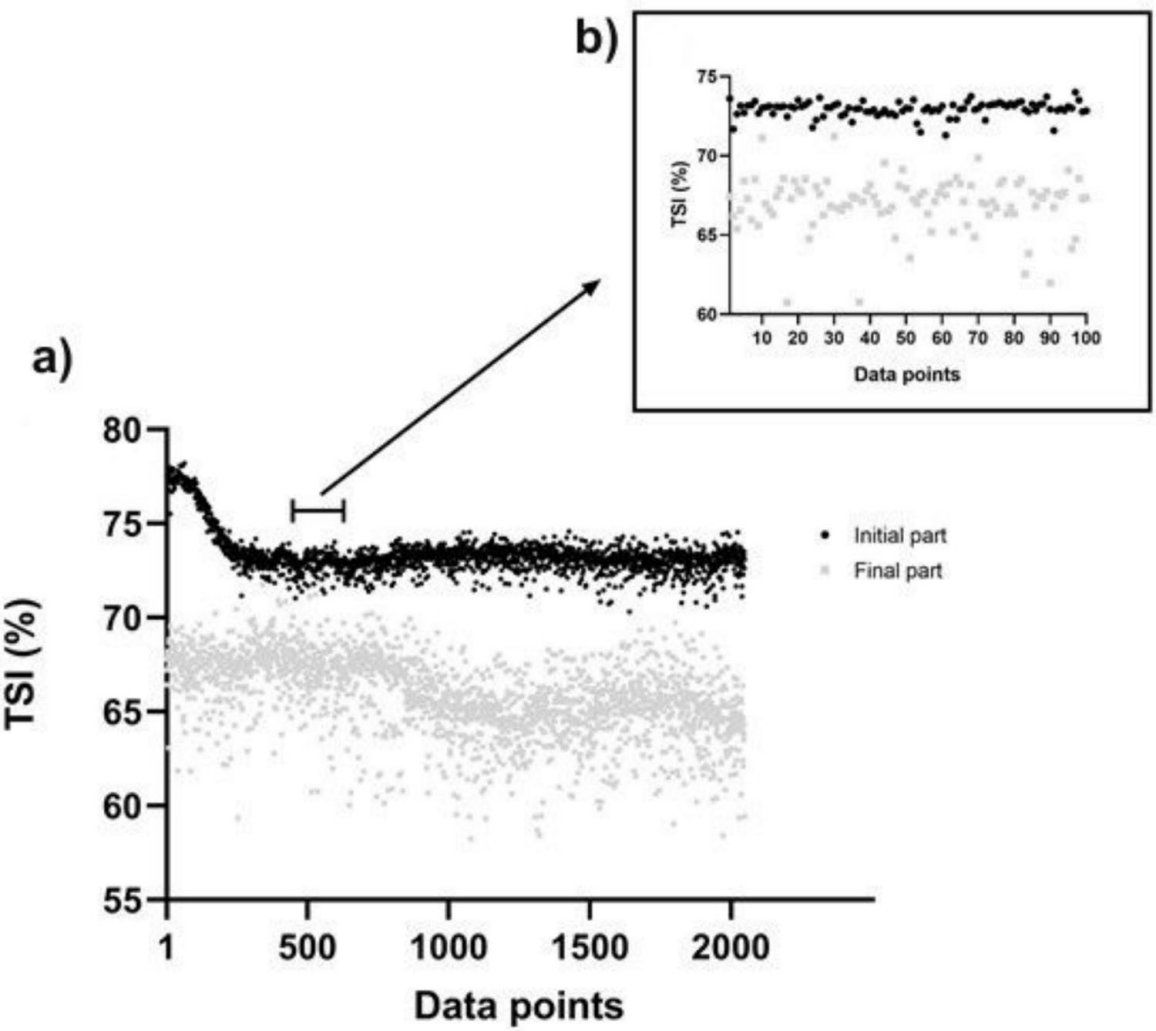
Example of raw Tissue saturation index (TSI) fluctuations at the initial (black, upper values) and final (grey, lower values) parts of the graded maximal exercise from one participant (a: the analyzed 2048 data points; b: zoom of 100 data points)

As illustrated in Fig. 3, with an example of one participant, the time-variability of TSI at the final part is larger compared to the initial. The fluctuations in the final part tended to be uncorrelated and random, resembling white noise. In contrast, the initial part exhibited more moderate persistence, characterized by a smoother pattern of tight increases and decreases.

At the end of the analysis, we introduced data shuffling after preprocessing to validate our findings. This randomization preserved the amplitude distribution of the signal while fully disrupting temporal correlations. As expected for uncorrelated white noise, the H-exponent in the shuffled data was consistent across both parts: 0.49 ± 0.03 (initial) and 0.49 ± 0.03 (final). In contrast, the original detrended signals from the initial part exhibited a substantially higher mean Hurst exponent of 0.84 ± 0.21, confirming the presence of long-range correlations.SampEn values were generally higher in the shuffled data, reflecting their greater unpredictability. In the final part, entropy changed only slightly after shuffling—from 1.40 to 1.57—suggesting that this part was already highly irregular following preprocessing.

## Discussion

This study, which aimed to investigate the effects of exercise intensity on the time-variability of muscle oxygen saturation, found that by the end of a graded maximal running exercise, the TSI fluctuations changed toward uncorrelated or disordered patterns, resembling random white noise. In contrast, at the beginning of the exercise, the TSI fluctuations were persistent and less variable, which can be related to a more tightly regulated response (Krstacic et al. 2002; Aoyagi et al. 2005; Gronwald et al. 2020; Van Hooren et al. 2023). These findings provided evidence that the time-variability structure of TSI was sensitive to the effects of the accumulated effort and increasing intensity during a graded running exercise.

Consistent with previous studies, the decrease in TSI observed at the end of the test likely reflects an imbalance between oxygen consumption and delivery. This reduction may be attributed to factors such as lowered pH, elevated lactate levels, lowered Hb-O_2_ affinity, increased temperature, or hemodynamic redistribution during high-intensity exercise (Mairbäurl 2013; Jones et al. 2015; Boezeman et al. 2016). Assessed indicators such as elevated HR and RPE, both associated with acute fatigue effects, further supported the presence of high-intensity effort. These results suggested that both the quantitative values of TSI and the qualitative changes in its fluctuations may complementary reflect aspects of metabolic strain and effort accumulation.

The observed tendency of the H-exponent approaching 0.5 and the increase in SampEn of TSI at the end of exercise align with physiological findings from previous studies on HRV, inter-breath intervals, and other cardiorespiratory variables in response to exercise effort (Krstacic et al. 2002; Garcia-Retortillo et al. 2017; Gronwald et al. 2019; Rogers et al. 2025) or to other stressors such as hypoxia and disease conditions (Scafetta et al. 2007). These results may reflect a reduction in the functional connectivity of regulatory mechanisms governing muscle oxygen concentration, likely involving inter-muscular, cardio-muscular, or cardio-respiratory networks (Balagué et al. 2016; Garcia-Retortillo et al. 2023; Garcia-Retortillo and Ivanov 2024).

Future research should offer a comprehensive network analysis of the time-variability structure of multilevel physiological variables, including muscle oxygen saturation, to elucidate their synergistic interrelations, potential time delays, and how these insights could collectively inform about the system's response to increasing workloads (Ivanov and Bartsch 2014; Balagué et al. 2020; Ivanov 2021). As there is a lack of available technology informing about the muscle metabolism dynamics during exercise, muscle oxygen saturation can contribute notably to the study of vertical network synergies across different organismic levels, such as tissues and organs (e.g., TSI and heart rate), occurring during exercise. Until now, only the dynamics of meso- and macroscopic variables such as muscle electrical activity, heart electrical activity, or accelerometry, integrating a broader range of physiological processes (e.g., metabolic, contractile, reflexive, volitional, among others), could help to provide an integrated assessment during exercise (Haken 1983; Balagué et al. 2020; Vázquez et al. 2021).

The temporal variability of muscle oxygen saturation may be particularly useful for detecting exercise intensity and related-fatigue effects during endurance activities, as it reflects underlying cardiorespiratory and metabolic demands (Mairbäurl 2013; Oueslati et al. 2016). While HRV has been widely applied to detect exercise workload in endurance activities (Gronwald et al. 2019; Mateo-March et al. 2023; Van Hooren et al. 2023; Rogers et al. 2025), its usefulness in resistance exercises remains less clear (Weippert et al. 2013; Kingsley and Figueroa 2016; Cauwenberghs et al. 2021). Thus, further research should evaluate the applicability of muscle oxygen saturation variability across different types of exercises. In addition, incorporating the intermediate phase of exhausting exercises—characterized by increased fluctuations as a transitional phase between stable and unstable states—may offer an alternative and potentially insightful approach for identifying critical points related to physiological thresholds in individual-specific contexts.

The sensitivity of muscle oxygen saturation time-variability to exercise intensity highlights its potential for developing monitoring tools to assess athlete’s health and performance. These tools could benefit from analyzing intra-individual variability of nonstationary time series rather than relying solely on tendencies, discrete values, or average distributions (Balagué et al. 2020). Time series analysis might offer a promising approach for detecting nonlinear events associated with exercise, including not only effort accumulation but also training effects (Garcia-Retortillo et al. 2017), task failure (Vázquez et al. 2016), overtraining (Tian et al. 2013; Armstrong et al. 2022), or injury risk (Pol et al. 2019; Fonseca et al. 2020). Early warning signals—reflected in alterations to the temporal structure of fluctuations, such as rising uncorrelated patterns—can reveal diminishing system adaptability and help anticipate critical transitions before adverse events occur (Scheffer et al. 2009). To fully realize this monitoring potential to practice, technological improvements are necessary in NIRS systems to ensure reliable signal acquisition and real-time variability analysis.

Future studies should address methodological limitations of this study and challenges on the reliability of NIRS, such as minimizing external noise effects (e.g., light interference or gait impact on muscle oxygen saturation), enhance light sources to increase tissue penetration, accounting for the influence of ventilatory work at high workloads (Thiel et al. 2011; Oueslati et al. 2016; Jeffries et al. 2019), as well as the effects of high skinfold thickness by particularly assessing the fat layers of individuals (Stuer et al. 2024). Additional NIRS-derived variables, such as oxyhemoglobin and deoxyhemoglobin, and relevant muscles during running such as gastrocnemius may also be included in analyses (Hiroyuki et al. 2002). Researchers are warranted to examine how time-variability in muscle oxygen saturation is affected by factors such as time series length, sampling frequency, exercise protocols and performance levels (Eke et al. 2012; Gronwald et al. 2020; Tuesta et al. 2024). Finally, advanced analytical methods, including Bayesian approaches for improving H-exponent estimation (Likens et al. 2023) and multifractal analyses (Kokosińska et al. 2018; Racz et al. 2018), should be considered to enhance the reliability and depth of time-variability assessments.

## Conclusions

The time-variability of muscle oxygen saturation shows promise as a sensitive indicator of exercise intensity during graded maximal running. The measure could serve as a valuable tool for the early detection of exhaustion and exercise tolerance, providing critical insights into individual responses to varying workloads. Further investigation is required to establish the time-variability of muscle oxygen saturation as a reliable and non-invasive measure for assessing exercise load and performance. Additionally, future research should explore muscle oxygen saturation to advance in the study of vertical network synergies during exercise.

## Conflict of interest

The authors declare no conflict of interest. This research did not receive any specific grant from funding agencies in the public, commercial, or not-for-profit sectors.

## Data Availability

All data acquired in the present study are available from the corresponding author upon reasonable request.

## Abbreviations

DFA: Detrended fluctuation analysis
H: Hurst exponent
HR: Heart rate
HRV: Heart rate variability
NIRS: Near-infrared spectroscopy
SampEn: Sample entropy
TSI: Tissue haemoglobin saturation index

## References

[CR1] Aoyagi N, Kiyono K, Struzik ZR, Yamamoto Y (2005) Changes in the hurst exponent of heart rate variability during physical activity. AIP Conf Proc 780:599–602. 10.1063/1.2036824

[CR2] Armstrong LE, Bergeron MF, Lee EC et al (2022) Overtraining syndrome as a complex systems phenomenon. Front Netw Physiol 1:1–20. 10.3389/fnetp.2021.794392 10.3389/fnetp.2021.794392 PMC10013019 36925581

[CR3] Balagué N, González J, Javierre C et al (2016) Cardiorespiratory coordination after training and detraining. a principal component analysis approach. Front Physiol 7:1–8. 10.3389/fphys.2016.00035 26903884 10.3389/fphys.2016.00035 PMC4751338

[CR4] Balagué N, Hristovski R, Almarcha M et al (2020) Network physiology of exercise: vision and perspectives. Front Physiol 11:1–18. 10.3389/fphys.2020.611550 33362584 10.3389/fphys.2020.611550 PMC7759565

[CR5] Balagué N, Hristovski R, Almarcha M et al (2022) Network physiology of exercise: beyond molecular and omics perspectives. Sports Med - Open 8:1–11. 10.1186/s40798-022-00512-0 36138329 10.1186/s40798-022-00512-0 PMC9500136

[CR6] Barstow TJ (2019) Understanding near infrared spectroscopy and its application to skeletal muscle research. J Appl Physiol 126:1360–1376. 10.1152/japplphysiol.00166.2018 30844336 10.1152/japplphysiol.00166.2018

[CR7] Batterson PM, Kirby BS, Hasselmann G, Feldmann A (2023) Muscle oxygen saturation rates coincide with lactate-based exercise thresholds. Eur J Appl Physiol 123:2249–2258. 10.1007/s00421-023-05238-9 37261552 10.1007/s00421-023-05238-9

[CR8] Bauer TA, Brass EP, Hiatt WR (2004) Impaired muscle oxygen use at onset of exercise in peripheral arterial disease. J Vasc Surg 40:488–493. 10.1016/j.jvs.2004.06.025 15337878 10.1016/j.jvs.2004.06.025

[CR9] Billat VL, Mille-Hamard L, Meyer Y, Wesfreid E (2009) Detection of changes in the fractal scaling of heart rate and speed in a marathon race. Physica A 388:3798–3808. 10.1016/j.physa.2009.05.029

[CR10] Boezeman RPE, Moll FL, Ünlü Ç, de Vries J-PPM (2016) Systematic review of clinical applications of monitoring muscle tissue oxygenation with near-infrared spectroscopy in vascular disease. Microvasc Res 104:11–22. 10.1016/j.mvr.2015.11.004 26576829 10.1016/j.mvr.2015.11.004

[CR11] Boone J, Barstow TJ, Celie B et al (2016) The interrelationship between muscle oxygenation, muscle activation, and pulmonary oxygen uptake to incremental ramp exercise: influence of aerobic fitness. Appl Physiol Nutr Metab 41:55–62. 10.1139/apnm-2015-0261 26701120 10.1139/apnm-2015-0261

[CR12] Borg G (1998) Borg’s perceived exertion and pain scales. Human Kinetics, Champaign, IL, US

[CR13] Cauwenberghs N, Cornelissen V, Christle JW et al (2021) Impact of age, sex and heart rate variability on the acute cardiovascular response to isometric handgrip exercise. J Hum Hypertens 35:55–64. 10.1038/s41371-020-0311-y 32042073 10.1038/s41371-020-0311-y

[CR14] Cohen J (1988) Statistical power analysis for the behavioral sciences. Erlbaum, Hillsdale, NJ

[CR15] Costa M, Goldberger AL, Peng C-K (2005) Multiscale entropy analysis of biological signals. Phys Rev E 71:021906. 10.1103/PhysRevE.71.021906 10.1103/PhysRevE.71.021906 15783351

[CR16] Crispin P, Forwood K (2021) Near infrared spectroscopy in anemia detection and management: a systematic review. Transfus Med Rev 35:22–28. 10.1016/j.tmrv.2020.07.003 32907764 10.1016/j.tmrv.2020.07.003

[CR17] Daniel N, Sybilski K, Kaczmarek W et al (2023) Relationship between EMG and fNIRS during dynamic movements. Sensors 23:5004. 10.3390/s23115004 37299730 10.3390/s23115004 PMC10255104

[CR18] Delgado-Bonal A, Marshak A (2019) Approximate entropy and sample entropy: a comprehensive tutorial. Entropy 21:541. 10.3390/e21060541 33267255 10.3390/e21060541 PMC7515030

[CR19] Desanlis J, Gordon D, French C et al (2024) Effects of occlusion pressure on hemodynamic responses recorded by near-infrared spectroscopy across two visits. Front Physiol 15:1–14. 10.3389/fphys.2024.1441239 10.3389/fphys.2024.1441239 PMC11422206 39324105

[CR20] Eke A, Herman P (1999) Fractal Analysis of Spontaneous Fluctuations in Human Cerebral Hemoglobin Content and its Oxygenation Level Recorded by NIRS. In: Eke A, Delpy DT (eds) Oxygen Transport to Tissue XXI. Springer, US, Boston, MA, pp 49–55 10.1007/978-1-4615-4717-4_7 10659131

[CR21] Eke A, Herman P, Sanganahalli BG et al (2012) Pitfalls in fractal time series analysis: fMRI BOLD as an exemplary case. Front Physiol 3:1–24. 10.3389/fphys.2012.00417 23227008 10.3389/fphys.2012.00417 PMC3513686

[CR22] Feldmann AM, Erlacher D, Pfister S, Lehmann R (2020) Muscle oxygen dynamics in elite climbers during finger-hang tests at varying intensities. Sci Rep 10:3040. 10.1038/s41598-020-60029-y 32080325 10.1038/s41598-020-60029-y PMC7033122

[CR23] Ferreira LF, Lutjemeier BJ, Townsend DK, Barstow TJ (2005) Dynamics of skeletal muscle oxygenation during sequential bouts of moderate exercise. Exp Physiol 90:393–401. 10.1113/expphysiol.2004.029595 15708875 10.1113/expphysiol.2004.029595

[CR24] Fonseca ST, Souza TR, Verhagen E et al (2020) sports injury forecasting and complexity: a synergetic approach. Sports Med 50:1757–1770. 10.1007/s40279-020-01326-4 32757162 10.1007/s40279-020-01326-4

[CR25] Fritz CO, Morris PE, Richler JJ (2012) Effect size estimates: Current use, calculations, and interpretation. J Exp Psychol Gen 141:2–18. 10.1037/a0024338 21823805 10.1037/a0024338

[CR26] Galaska R, Makowiec D, Dudkowska A et al (2008) Comparison of wavelet transform modulus maxima and multifractal detrended fluctuation analysis of heart rate in patients with systolic dysfunction of left ventricle. Ann Noninvasive Electrocardiol 13:155–164. 10.1111/j.1542-474X.2008.00215.x 18426441 10.1111/j.1542-474X.2008.00215.x PMC6932668

[CR27] Garcia-Retortillo S, Ivanov PC (2024) Dynamics of cardio-muscular networks in exercise and fatigue. J Physiol. 10.1113/JP286963 39392864 10.1113/JP286963

[CR28] Garcia-Retortillo S, Javierre C, Hristovski R et al (2017) Cardiorespiratory coordination in repeated maximal exercise. Front Physiol 8:1–7. 10.3389/fphys.2017.00387 28638349 10.3389/fphys.2017.00387 PMC5461287

[CR29] Garcia-Retortillo S, Romero-Gómez C, Ivanov PC (2023) Network of muscle fibers activation facilitates inter-muscular coordination, adapts to fatigue and reflects muscle function. Commun Biol 6:1–22. 10.1038/s42003-023-05204-3 37648791 10.1038/s42003-023-05204-3 PMC10468525

[CR30] Goldberger AL, West BJ (1987) Fractals in physiology and medicine. Yale J Biol Med 60:421–435 3424875 PMC2590346

[CR31] Gronwald T, Hoos O (2020) Correlation properties of heart rate variability during endurance exercise: a systematic review. Ann Noninvasive Electrocardiol 25:e12697. 10.1111/anec.12697 31498541 10.1111/anec.12697 PMC7358842

[CR32] Gronwald T, Hoos O, Ludyga S, Hottenrott K (2019) Non-linear dynamics of heart rate variability during incremental cycling exercise. Res Sports Med 27:88–98. 10.1080/15438627.2018.1502182 30040499 10.1080/15438627.2018.1502182

[CR33] Gronwald T, Rogers B, Hoos O (2020) Fractal correlation properties of heart rate variability: a new biomarker for intensity distribution in endurance exercise and training prescription? Front Physiol 11:1–11. 10.3389/fphys.2020.550572 33071812 10.3389/fphys.2020.550572 PMC7531235

[CR34] Haken H (1983) Synergetics: An Introduction. Springer, Berlin, Heidelberg

[CR35] Hardstone R, Poil S-S, Schiavone G et al (2012) Detrended fluctuation analysis: a scale-free view on neuronal oscillations. Front Physiol 3:1–13. 10.3389/fphys.2012.00450 23226132 10.3389/fphys.2012.00450 PMC3510427

[CR36] Hautala AJ, Mäkikallio TH, Seppänen T et al (2003) Short-term correlation properties of R-R interval dynamics at different exercise intensity levels. Clin Physiol Funct Imaging 23:215–223. 10.1046/j.1475-097X.2003.00499.x 12914561 10.1046/j.1475-097x.2003.00499.x

[CR37] Hesford C, Cardinale M, Laing S, Cooper CE (2013) NIRS Measurements with Elite Speed Skaters: Comparison Between the Ice Rink and the Laboratory. In: Welch WJ, Palm F, Bruley DF, Harrison DK (eds) Oxygen Transport to Tissue XXXIV. Springer, New York, pp 81–86 10.1007/978-1-4614-4989-8_12 22879018

[CR38] Hiroyuki H, Hamaoka T, Sako T et al (2002) Oxygenation in vastus lateralis and lateral head of gastrocnemius during treadmill walking and running in humans. Eur J Appl Physiol 87:343–349. 10.1007/s00421-002-0644-y 12172872 10.1007/s00421-002-0644-y

[CR39] Hong Y, Bao D, Manor B et al (2024) Effects of endurance exercise on physiologic complexity of the hemodynamics in prefrontal cortex. Nph 11:015009. 10.1117/1.NPh.11.1.015009 10.1117/1.NPh.11.1.015009 PMC10956706 38515930

[CR40] Hunter B, Karsten B, Greenhalgh A et al (2023) The application of non-linear methods to quantify changes to movement dynamics during running: a scoping review. J Sports Sci 41:481–494. 10.1080/02640414.2023.2225014 37330658 10.1080/02640414.2023.2225014

[CR41] Ihlen EAF (2012) Introduction to multifractal detrended fluctuation analysis in Matlab. Front Physiol 3:1–18. 10.3389/fphys.2012.00141 22675302 10.3389/fphys.2012.00141 PMC3366552

[CR42] Ivanov PC (2021) The new field of network physiology: building the human physiolome. Front Netw Physiol 1:1–15. 10.3389/fnetp.2021.711778 10.3389/fnetp.2021.711778 PMC10013018 36925582

[CR43] Ivanov PC, Bartsch RP (2014) Network Physiology: Mapping Interactions Between Networks of Physiologic Networks. In: D’Agostino G, Scala A (eds) Networks of Networks: The Last Frontier of Complexity. Springer International Publishing, Cham, pp 203–222

[CR44] Jeffries O, Patterson SD, Waldron M (2019) The effect of severe and moderate hypoxia on exercise at a fixed level of perceived exertion. Eur J Appl Physiol 119:1213–1224. 10.1007/s00421-019-04111-y 30820661 10.1007/s00421-019-04111-y PMC6469630

[CR45] Jiang Y, Costello JT, Williams TB et al (2021) A network physiology approach to oxygen saturation variability during normobaric hypoxia. Exp Physiol 106:151–159. 10.1113/EP088755 32643311 10.1113/EP088755

[CR46] Jones B, Dat M, Cooper CE (2014) Underwater near-infrared spectroscopy measurements of muscle oxygenation: laboratory validation and preliminary observations in swimmers and triathletes. JBO 19:127002. 10.1117/1.JBO.19.12.127002 25478871 10.1117/1.JBO.19.12.127002

[CR47] Jones B, Hamilton DK, Cooper CE (2015) Muscle oxygen changes following sprint interval cycling training in elite field hockey players. PLoS ONE 10:e0120338. 10.1371/journal.pone.0120338 25807517 10.1371/journal.pone.0120338 PMC4373931

[CR48] Jones S, Chiesa ST, Chaturvedi N, Hughes AD (2016) Recent developments in near-infrared spectroscopy (NIRS) for the assessment of local skeletal muscle microvascular function and capacity to utilise oxygen. Artery Research 16:25–33. 10.1016/j.artres.2016.09.001 27942271 10.1016/j.artres.2016.09.001 PMC5134760

[CR49] Kantelhardt JW, Zschiegner SA, Koscielny-Bunde E et al (2002) Multifractal detrended fluctuation analysis of nonstationary time series. Physica A 316:87–114. 10.1016/S0378-4371(02)01383-3

[CR50] Kerhervé HA, McLean S, Birkenhead K et al (2017) Influence of exercise duration on cardiorespiratory responses, energy cost and tissue oxygenation within a 6 hour treadmill run. PeerJ 5:e3694. 10.7717/peerj.3694 29038746 10.7717/peerj.3694 PMC5637745

[CR51] Kingsley JD, Figueroa A (2016) Acute and training effects of resistance exercise on heart rate variability. Clin Physiol Funct Imaging 36:179–187. 10.1111/cpf.12223 25524332 10.1111/cpf.12223

[CR52] Klusiewicz A, Rębiś K, Ozimek M, Czaplicki A (2021) The use of muscle near-infrared spectroscopy (NIRS) to assess the aerobic training loads of world-class rowers. Biol Sport 38:713–719. 10.5114/biolsport.2021.103571 34937982 10.5114/biolsport.2021.103571 PMC8670802

[CR53] Kokosińska D, Gierałtowski JJ, Żebrowski JJ et al (2018) Heart rate variability, multifractal multiscale patterns and their assessment criteria. Physiol Meas 39:114010. 10.1088/1361-6579/aae86d 30485251 10.1088/1361-6579/aae86d

[CR54] Kosciessa JQ, Kloosterman NA, Garrett DD (2020) Standard multiscale entropy reflects neural dynamics at mismatched temporal scales: What’s signal irregularity got to do with it? PLoS Comput Biol 16:e1007885. 10.1371/journal.pcbi.1007885 32392250 10.1371/journal.pcbi.1007885 PMC7241858

[CR55] Krstacic G, Krstacic A, Martinis M, et al (2002) Non-linear analysis of heart rate variability in patients with coronary heart disease. In: Computers in Cardiology. pp 673–675

[CR56] Lake DE, Moorman JR (2011) Accurate estimation of entropy in very short physiological time series: the problem of atrial fibrillation detection in implanted ventricular devices. Am J Physiol-Heart Circulatory Physiol 300:H319–H325. 10.1152/ajpheart.00561.2010 10.1152/ajpheart.00561.2010 21037227

[CR57] Lewis MJ, Short AL (2007) Sample entropy of electrocardiographic RR and QT time-series data during rest and exercise. Physiol Meas 28:731. 10.1088/0967-3334/28/6/011 17664626 10.1088/0967-3334/28/6/011

[CR58] Likens AD, Mangalam M, Wong AY, et al (2023) Better than DFA? A Bayesian Method for Estimating the Hurst Exponent in Behavioral Sciences. ArXiv arXiv:2301.11262v1

[CR59] Lucero AA, Addae G, Lawrence W et al (2018) Reliability of muscle blood flow and oxygen consumption response from exercise using near-infrared spectroscopy. Exp Physiol 103:90–100. 10.1113/EP086537 29034529 10.1113/EP086537 PMC12884174

[CR60] Mairbäurl H (2013) Red blood cells in sports: Effects of exercise and training on oxygen supply by red blood cells. Front Physiol 4:1–13. 10.3389/fphys.2013.00332 24273518 10.3389/fphys.2013.00332 PMC3824146

[CR61] Manchado-Gobatto FB, Marostegan AB, Rasteiro FM et al (2020) New insights into mechanical, metabolic and muscle oxygenation signals during and after high-intensity tethered running. Sci Rep 10:6336. 10.1038/s41598-020-63297-w 32286408 10.1038/s41598-020-63297-w PMC7156678

[CR62] Martin DS, Levett DZ, Mythen M et al (2009) Changes in skeletal muscle oxygenation during exercise measured by near-infrared spectroscopy on ascent to altitude. Crit Care 13:S7. 10.1186/cc8005 19951391 10.1186/cc8005 PMC2786109

[CR63] Martinis M, Knežević A, Krstačić G, Vargović E (2004) Changes in the Hurst exponent of heartbeat intervals during physical activity. Phys Rev E 70:012903. 10.1103/PhysRevE.70.012903 10.1103/PhysRevE.70.012903 15324105

[CR64] Mateo-March M, Moya-Ramón M, Javaloyes A et al (2023) Validity of detrended fluctuation analysis of heart rate variability to determine intensity thresholds in elite cyclists. Eur J Sport Sci 23:580–587. 10.1080/17461391.2022.2047228 35238695 10.1080/17461391.2022.2047228

[CR65] Mesquita RC, Putt ME, Chandra M et al (2013) Diffuse optical characterization of an exercising patient group with peripheral artery disease. JBO 18:057007. 10.1117/1.JBO.18.5.057007 23708193 10.1117/1.JBO.18.5.057007 PMC3662991

[CR66] Montull L, Matas S, Canton A et al (2025) Novel possibilities of acceleration time series for performance and acute fatigue assessment in uphill trail running. Sports Biomech. 10.1080/14763141.2025.2486088 40207655 10.1080/14763141.2025.2486088

[CR67] Oueslati F, Boone J, Ahmaidi S (2016) Respiratory muscle endurance, oxygen saturation index in vastus lateralis and performance during heavy exercise. Respir Physiol Neurobiol 227:41–47. 10.1016/j.resp.2016.02.008 26923271 10.1016/j.resp.2016.02.008

[CR68] Peng CK, Buldyrev SV, Havlin S et al (1994) Mosaic organization of DNA nucleotides. Phys Rev E 49:1685–1689. 10.1103/PhysRevE.49.1685 10.1103/physreve.49.1685 9961383

[CR69] Peng CK, Havlin S, Stanley HE, Goldberger AL (1995) Quantification of scaling exponents and crossover phenomena in nonstationary heartbeat time series. Chaos: an Interdisciplinary J Nonlinear Sci 5:82–87. 10.1063/1.166141 10.1063/1.166141 11538314

[CR70] Perrey S (2022) Muscle oxygenation unlocks the secrets of physiological responses to exercise: time to exploit it in the training monitoring. Front Sports Act Living 4:1–4. 10.3389/fspor.2022.864825 10.3389/fspor.2022.864825 PMC8936169 35321522

[CR71] Perrey S, Quaresima V, Ferrari M (2024) Muscle oximetry in sports science: an updated systematic review. Sports Med 54:975–996. 10.1007/s40279-023-01987-x 38345731 10.1007/s40279-023-01987-x PMC11052892

[CR72] Pethick J, Winter SL, Burnley M (2021) Physiological complexity: influence of ageing, disease and neuromuscular fatigue on muscle force and torque fluctuations. Exp Physiol 106:2046–2059. 10.1113/EP089711 34472160 10.1113/EP089711

[CR73] Pol R, Hristovski R, Medina D, Balague N (2019) From microscopic to macroscopic sports injuries. applying the complex dynamic systems approach to sports medicine: a narrative review. Br J Sports Med 53:1214–1220. 10.1136/bjsports-2016-097395 29674346 10.1136/bjsports-2016-097395

[CR74] Racz FS, Stylianou O, Mukli P, Eke A (2018) Multifractal dynamic functional connectivity in the resting-state brain. Front Physiol. 10.3389/fphys.2018.01704 30555345 10.3389/fphys.2018.01704 PMC6284038

[CR75] Richman JS, Moorman JR (2000) Physiological time-series analysis using approximate entropy and sample entropy. Am J Physiol-Heart Circulatory Physiol 278:H2039–H2049. 10.1152/ajpheart.2000.278.6.H2039 10.1152/ajpheart.2000.278.6.H2039 10843903

[CR76] Riganello F, Larroque SK, Bahri MA et al (2018) A heartbeat away from consciousness: heart rate variability entropy can discriminate disorders of consciousness and is correlated with resting-State fMRI brain connectivity of the central autonomic network. Front Neurol 9:1–18. 10.3389/fneur.2018.00769 30258400 10.3389/fneur.2018.00769 PMC6145008

[CR77] Rogers B, Fleitas-Paniagua PR, Trpcic M et al (2025) Fractal correlation properties of heart rate variability and respiratory frequency as measures of endurance exercise durability. Eur J Appl Physiol. 10.1007/s00421-025-05716-2 39904800 10.1007/s00421-025-05716-2

[CR78] Scafetta N, Moon RE, West BJ (2007) Fractal response of physiological signals to stress conditions, environmental changes, and neurodegenerative diseases. Complexity 12:12–17. 10.1002/cplx.20183

[CR79] Scheffer M, Bascompte J, Brock WA et al (2009) Early-warning signals for critical transitions. Nature 461:53–59. 10.1038/nature08227 19727193 10.1038/nature08227

[CR80] Sendra-Pérez C, Sanchez-Jimenez JL, Marzano-Felisatti JM et al (2023) Reliability of threshold determination using portable muscle oxygenation monitors during exercise testing: a systematic review and meta-analysis. Sci Rep 13:12649. 10.1038/s41598-023-39651-z 37542055 10.1038/s41598-023-39651-z PMC10403529

[CR81] Stuer L, Teso M, Colosio AL et al (2024) The impact of skinfold thickness and exercise intensity on the reliability of NIRS in the vastus lateralis. Eur J Appl Physiol. 10.1007/s00421-024-05654-5 39572450 10.1007/s00421-024-05654-5

[CR82] Thiel C, Vogt L, Himmelreich H et al (2011) Reproducibility of muscle oxygen saturation. Int J Sports Med 32:277–280. 10.1055/s-0030-1269922 21271493 10.1055/s-0030-1269922

[CR83] Tian Y, He ZH, Zhao JX et al (2013) Heart rate variability threshold values for early-warning nonfunctional overreaching in elite female wrestlers. J Strength Cond Res 27:1511–1519. 10.1519/JSC.0b013e31826caef8 23715265 10.1519/JSC.0b013e31826caef8

[CR84] Tuesta M, Yáñez-Sepúlveda R, Monsalves-Álvarez M et al (2024) Muscle oxygen extraction during vascular occlusion test in physically very active versus inactive healthy men: a comparative study. J Functional Morphol Kinesiol 9:57. 10.3390/jfmk9020057 10.3390/jfmk9020057 PMC10961797 38525758

[CR85] Van Hooren B, Bongers BC, Rogers B, Gronwald T (2023) The between-day reliability of correlation properties of heart rate variability during running. Appl Psychophysiol Biofeedback 48:453–460. 10.1007/s10484-023-09599-x 37516677 10.1007/s10484-023-09599-x PMC10582140

[CR86] van Rassel CR, Ajayi OO, Sales KM et al (2025) Quantifying exercise intensity with fractal correlation properties of heart rate variability: a study on incremental and constant-speed running. Eur J Appl Physiol 125:91–102. 10.1007/s00421-024-05592-2 39235602 10.1007/s00421-024-05592-2

[CR87] Vázquez P, Hristovski R, Balagué N (2016) The path to exhaustion: time-variability properties of coordinative variables during continuous exercise. Front Physiol 7:1–8. 10.3389/fphys.2016.00037 26913006 10.3389/fphys.2016.00037 PMC4753307

[CR88] Vázquez P, Petelczyc M, Hristovski R, Balagué N (2021) Interlimb coordination: a new order parameter and a marker of fatigue during quasi-isometric exercise? Front Physiol 11:1–12. 10.3389/fphys.2020.612709 10.3389/fphys.2020.612709 PMC7835426 33510649

[CR89] Weippert M, Behrens K, Rieger A et al (2013) Heart rate variability and blood pressure during dynamic and static exercise at similar heart rate levels. PLoS ONE 8:e83690. 10.1371/journal.pone.0083690 24349546 10.1371/journal.pone.0083690 PMC3862773

[CR90] Weippert M, Behrens M, Rieger A, Behrens K (2014) Sample entropy and traditional measures of heart rate dynamics reveal different modes of cardiovascular control during low intensity exercise. Entropy 16:5698–5711. 10.3390/e16115698

[CR91] Yogev A, Arnold J, Nelson H et al (2023) Comparing the reliability of muscle oxygen saturation with common performance and physiological markers across cycling exercise intensity. Front Sports Act Living 5:1–10. 10.3389/fspor.2023.1143393 10.3389/fspor.2023.1143393 PMC10436610 37601168

[CR92] Zhou J, Manor B, Liu D et al (2013) The complexity of standing postural control in older adults: a modified detrended fluctuation analysis based upon the empirical mode decomposition algorithm. PLoS ONE 8:e62585. 10.1371/journal.pone.0062585 23650518 10.1371/journal.pone.0062585 PMC3641070

[CR93] Zorgati H, Collomp K, Boone J et al (2015) Effect of pedaling cadence on muscle oxygenation during high-intensity cycling until exhaustion: a comparison between untrained subjects and triathletes. Eur J Appl Physiol 115:2681–2689. 10.1007/s00421-015-3235-4 26255290 10.1007/s00421-015-3235-4

